# Exosomes derived from umbilical cord mesenchymal stem cells alleviate viral myocarditis through activating AMPK/mTOR‐mediated autophagy flux pathway

**DOI:** 10.1111/jcmm.15378

**Published:** 2020-05-18

**Authors:** Xiaohong Gu, Yuechun Li, Kaixin Chen, Xingang Wang, Zhongyu Wang, Hao Lian, Yuanzheng Lin, Xing Rong, Maoping Chu, Jiafeng Lin, Xiaoling Guo

**Affiliations:** ^1^ Institute of Cardiovascular Development and Translational Medicine The Second Affiliated Hospital and Yuying Children’s Hospital of Wenzhou Medical University Wenzhou China; ^2^ Center of Scientific Research The Second Affiliated Hospital and Yuying Children’s Hospital of Wenzhou Medical University Wenzhou China

**Keywords:** AMPK, apoptosis, exosomes, human umbilical cord mesenchymal stem cells, mTOR‐mediated autophagy flux, viral myocarditis

## Abstract

Human umbilical cord mesenchymal stem cell‐derived exosomes (hucMSC‐exosomes) have been implicated as a novel therapeutic approach for tissue injury repair and regeneration, but the effects of hucMSC‐exosomes on coxsackievirus B3 (CVB3)‐induced myocarditis remain unknown. The object of the present study is to investigate whether hucMSC‐exosomes have therapeutic effects on CVB3‐induced myocarditis (VMC). HucMSC‐exosomes were identified using nanoparticle tracking analysis (NTA), transmission electron microscopy (TEM) and Western blot. The purified hucMSC‐exosomes tagged with PKH26 were tail intravenously injected into VMC model mice in vivo and used to administrate CVB3‐infected human cardiomyocytes (HCMs) in vitro, respectively. The effects of hucMSC‐exosomes on myocardial pathology injury, proinflammatory cytokines and cardiac function were evaluated through haematoxylin and eosin (H&E) staining, quantitative polymerase chain reaction (qPCR) and Doppler echocardiography. The anti‐apoptosis role and potential mechanism of hucMSC‐exosomes were explored using TUNEL staining, flow cytometry, immunohistochemistry, Ad‐mRFP‐GFP‐LC3 transduction and Western blot. In vivo results showed that hucMSC‐exosomes (50 μg iv) significantly alleviated myocardium injury, shrank the production of proinflammatory cytokines and improved cardiac function. Moreover, in vitro data showed that hucMSC‐exosomes (50 μg/mL) inhibited the apoptosis of CVB3‐infected HCM through increasing pAMPK/AMPK ratio and up‐regulating autophagy proteins LC3II/I, BECLIN‐1 and anti‐apoptosis protein BCL‐2 as well as decreasing pmTOR/mTOR ratio, promoting the degradation of autophagy flux protein P62 and down‐regulating apoptosis protein BAX. In conclusion, hucMSC‐exosomes could alleviate CVB3‐induced myocarditis via activating AMPK/mTOR‐mediated autophagy flux pathway to attenuate cardiomyocyte apoptosis, which will be benefit for MSC‐exosome therapy of myocarditis in the future.

## 1 INTRODUCTION

Viral myocarditis (VMC) is an important cause of heart failure, arrhythmia and sudden death in young people, which is related to immune injure, autophagy and apoptosis induced by viral infection.^1^, ^2^ In the past decades, myocarditis patients had partial or full clinical recovery with conventional medical treatment, immunomodulatory therapy and immunosuppressive therapy, but those who did not be recovered might develop into dilated cardiomyopathy.^3^, ^4^, ^5^, ^6^ Therefore, exploring effective and novel therapy methods is becoming more necessary. Mesenchymal stem cells (MSCs) derived from different tissues including bone marrow, adipose and umbilical cord are commonly used in tissue repair and regeneration.^7^, ^8^, ^9^ The transfusion of MSCs derived from bone marrow, adipose and umbilical cord showed therapeutic effects on type 2 diabetes rat models and even patients.^10^, ^11^, ^12^, ^13^ Intramyocardial, intramuscular and intravenous injections of MSCs have shown to improve cardiac function in myocardial infarction pigs, dilated cardiomyopathy rats and chronic myocardial ischaemia pigs.^14^, ^15^, ^16^ Human umbilical cord MSCs (hucMSCs) have low immunogenicity, high self‐renewal ability and fewer ethical issues, so they are more suitable for clinical application.^17^, ^18^ Although playing a positive therapeutic effect, MSCs often do not persist or integrate in tissues.^19^

Recently, the study had demonstrated that MSCs through paracrine pathway play a major role in tissue repair and regeneration.^20^ The soluble paracrine factors can promote angiogenesis and myocardial regeneration, and can also recruit cardiac stem cells or bone marrow cells into the niche of myocardial injury.^21^, ^22^ Since the complexity and diversity of the paracrine factors, it is hard to identify which paracrine factor plays crucial role in the therapy. Timmers et al reported that myocardial ischaemia/reperfusion injury could be repaired by factors with molecular weight greater than 1000 kD in human embryonic MSC medium, and then, they further confirmed these factors were exosomes derived from MSCs.^23^ The function of exosomes derived from MSCs had been investigated in many diseases such as myocardial ischaemia, ischaemia/reperfusion, autoimmune and neurodegenerative disorders, cancer and diabetes.^24^, ^25^, ^26^, ^27^, ^28^

Exosomes, having the diameter of 30‐150 nm, are the efficient component of paracellular secretion and play the important roles in intercellular communication.^19^, ^29^, ^30^, ^31^ Therefore, exosomes will be the great potential therapeutic agent to replace stem cells in reducing tissue injury and enhancing tissue repair.

However, whether hucMSC‐exosomes can rescue myocardial damage and improve cardiac function in myocarditis is still unknown. Thus, in this study, we will investigate whether hucMSC‐exosomes can play a protective role in the coxsackievirus B3 (CVB3)‐induced myocarditis and explore the underlying mechanisms.

## 2 MATERIALS AND METHODS

### 2.1 Isolation and culture human umbilical cord mesenchymal stem cells (hucMSCs)

Human umbilical cords were obtained from full‐term newborn infants by caesarean section in the Second Affiliated Hospital and Yuying Children's Hospital of Wenzhou Medical University. The informed consent was acquired from every donor parent, and this study was approved by Human Research and Ethical Committee of Wenzhou Medical University. Human umbilical cord mesenchymal stem cells (hucMSCs) were obtained using collagenase digestion. Briefly, under the sterile condition, umbilical cord tissues were cut open, and then, the blood vessels were removed. The umbilical cord tissues were washed twice with fresh phosphate buffer solution (PBS, Gibco, Grand Island, NY, USA) containing 1% gentamycin to remove blood clots and red blood cells, and then were dissected into 1 mm^3^ pieces. Then, they were digested using 0.1% type II collagenase (Sigma, St. Louis, MO, USA) at 37°C shaking table for 6 hours and were filtered using a 100‐mesh sieve. The filtrate was centrifuged at 1000 rpm for 10 minutes and wished twice with PBS. The sediments were suspended using MSC medium containing Dulbecco's Modified Eagle's Medium/F12 (DMEM/F12, Gibco), 10% foetal bovine serum (FBS, Gibco) and 1% penicillin and streptomycin (P/S, Gibco), and then were transferred into the 25 cm^2^ Petri dishes at a 37°C, 5% CO_2_ incubator. The medium was replaced with fresh medium every 2 days. When reaching 80%‐90% confluence, the cells would be passaged with 0.25% EDTA‐trypsin.

### 2.2 Flow cytometry

Flow cytometry was performed as the previous report.^18^ Briefly, the third passage hucMSCs were harvested using 0.25% trypsin and washed twice using PBS. Then, hucMSCs were fixed using 4% paraformaldehyde (Sigma) and incubated with direct fluorescent label antibodies or isotype control antibodies as the Table S1 at 4°C for 30 min. The samples were detected by flow cytometry analyzer (BD, USA).

### 2.3 Isolation and identification of exosomes

After reaching 80% confluence, hucMSCs were washed three times with PBS, and then, the medium were changed with DMEM/F12 containing 10% exosome‐depleted FBS, which was prepared through ultracentrifugation at 120 000 *g* at 4°C for 12 hours. After 48 hours, the cultured medium was harvested and centrifuged at 300 *g* for 10 minutes, 2000 *g* for 10 minutes and 10 000 *g* for 50 minutes separately to remove floating cells and cellular debris. Then, the harvested medium was filtered using the 0.22‐μm filter. Then, the medium was ultracentrifugation at 120 000 *g* for 90 minutes to get the exosomes, and the harvested exosomes were washed once using PBS. Finally, the harvested exosomes were resuspended in PBS and stored at −80°C. Meanwhile, nanoparticle tracking analysis (NTA) was performed to detected size distribution, and the concentration of exosomes was measured using ZetaView (Particle Metrix, Germany). The morphology of exosomes was also observed under a transmission electron microscopy (TEM, Japan). The bicinchoninic acid (BCA) protein assay kit (Beyotime, China) was used to measure the protein concentration of exosomes isolated from the cultured medium of hucMSCs according the previous reports,^24^, ^32^ which was used to relatively quantify the concentration of exosomes. The expressions of exosome protein markers such as CD81, CD63 and CD9 were analysed by Western blot, and the information of these antibodies was shown in the Table S2.

### 2.4 Virus

Coxsackievirus B3 (CVB3, Nancy strain, USA) virus was maintained and expanded using Hep2 cells. The viral titre was determined through the 50% tissue culture infectious dose (TCID50) assay.

### 2.5 Animal experiment

All animal experiments were followed with the Guide for the Care and Use of Laboratory Animals by the National Institutes of Health and were approved by the Animal Ethics Committee of Wenzhou Medical University. Thirty‐five‐day‐old male BALB/c mice (20‐22 g) were purchased from the SLAC Laboratory Animal Center of Shanghai. They were fed in a 12‐h dark/light cycle under temperature at 23 ± 2°C, with ad libitum access to food and water. BALB/c mice were intraperitoneally injected with 0.1 mL of normal saline (NS) containing 1 × 10^5^ TCID50 of CVB3 to establish the mouse VMC model. The control group was intraperitoneally injected with 0.1 mL NS without CVB3. The mice were randomly divided into four groups namely the control group (0.1 mL NS iv), VMC group (0.1 mL NS iv), VMC + L‐EXO group (0.1 mL NS with 5 μg hucMSC‐exosomes iv) and VMC + H‐EXO group (0.1 mL NS with 50 μg hucMSC‐exosomes iv).

### 2.6 Cell experiment

Human cardiomyocytes sv40 (HCM, Abmgood, USA) were cultured in Prigrow I medium (Abmgood) with 10% exosome‐depleted FBS at 37°C, 5% CO_2_ incubator. The medium was refreshed every 2 days. HCMs were also exposed to CVB3 at a multiplication of infection (MOI) of 10 for 2 hours under FBS‐starvation condition to establish the cell VMC model. The control group was just treated with FBS‐free medium for 2 hours. For exploring the underlying mechanisms, cells at the density of 3 × 10^5^ cells/well were seeded into six‐well plates and incubated for 24 hours; then, the cells in VMC model were treated with exosomes (50 μg/mL) and supplemented with AMPK inhibitor compound C (CC, 10 μmol/L, MedChem Express Biotechnology, Monmouth Junction, NJ, USA) or autophagy agonist rapamycin (RAPA, 20 nmol/L, Sigma‐Aldrich, St. Louis, MO, USA) according to the specific demands for next 24 hours. At last, the treated cells were used for following experiments.

### 2.7 Uptake of PKH26‐labelled hucMSC‐exosomes

Exosomes derived from hucMSCs (hucMSC‐exosomes) were labelled with PKH26 (Sigma‐Aldrich, USA) according to manufacturer's instructions. Briefly, 1 μL PKH26 was mixed with 250 μL Diluent C. Then, 25 μL hucMSC‐exosomes were added into the mixture and incubated at room temperature (RT) for 5 minutes. Then, 500 μL 0.5% exosome‐depleted FBS was added and incubated at RT for 5 minutes to stop the reaction. Then, exosomes were centrifuged at 120 000 *g* for 90 minutes at 4°C and resuspended in PBS prior to the uptake assay.

For the animal uptake assay, mice were injected with PKH26 labelled exosomes (5 μg or 50 μg) or the equal volume of PBS as control through tail vein. After treatment, the mice were killed, and the heart tissues were collected and frozen using optimal cutting temperature compound at −80°C on day 7. The frozen tissues were continuously cut into 5 μm slices. For the cell uptake assay, HCMs were incubated with 50 μg/mL PKH26 labelled exosomes or the equal volume of PBS as control in the medium containing 10% exosome‐depleted FBS for 24 hours at 37°C, 5% CO_2_ incubator.

The frozen sections and cells were washed with PBS twice, fixed with freshly 4% paraformaldehyde at RT for 10 minutes and permeabilized in 0.1% triton X‐100/PBS at RT for 15 minutes. Then, they were blocked with 3% BSA for 1 hour at 25°C and were incubated with anti‐cardiac troponin I (cTNI) antibody for 12 hours at 4°C. Then, they were stained with the 1:300 dilution of secondary antibody Alexa Flour^®^ 488‐conjugated goat anti‐rabbit IgG (Abcam, Cambridge, MA, USA) for 1 hour at 37°C and 4’, 6‐diamidine‐2’2phenylindole dihydrochloride (DAPI) (Beyotime, Songjiang, Shanghai, China) for 5 minutes at RT. Lastly, they were observed under fluorescence microscopy (Olympus, Shinjuku, Tokyo, Japan).

### 2.8 Assessment of pathological changes

On day 7, the hearts of mice from each group were fixed with 4% formalin, dehydrated, embedded in paraffin and cut into 5 μm sections. Pathological changes in heart tissues were visualized using haematoxylin and eosin (H&E) staining. Each heart was cut into an average of 3 slices, and each slice was used to observe 5 visual fields. Semi‐quantitative scoring of inflammation, oedema and necrosis of heart tissues was performed under the optical microscopy examination. The degree of heart injury was blindly graded by two independent observers as described previously.^33^ The pathological score was graded as follows: a score of 0 indicated no lesion; a score of 1 indicated lesion involving 25% of the myocardium; a score of 2 indicated lesions involving 25% to 50% of the myocardium; a score of 3 indicated lesions involving 50% to 75% of the myocardium; and a score of 4 indicated lesions involving 75% to 100% of the myocardium. The scores for every section were averaged.

### 2.9 Real‐time quantitative PCR (RT‐qPCR)

Total RNAs were extracted from heart tissues of four groups using TRIzol Reagent (Invitrogen, Carlsbad, CA, USA), and then, they were reverse‐transcribed into cDNA with TransScript II one‐step gDNA removal and cDNA Synthesis SuperMix (TransGen, Haidian, Beijing, China) according to manufacturer's instructions. These cDNAs were used as the templates for subsequent qPCR measurement with IQ SYBR Green SuperMix (Bio‐Rad, Berkeley, CA, USA). The levels of mRNA (IL‐1, IL‐6, TNF‐α and ATG5) expressions were calculated using the 2^(−ΔΔCT)^ method and normalized against GAPDH levels. All reactions were run in triplicate. The primer sequences were listed in the Table S3.

### 2.10 Doppler echocardiography study

Transthoracic echocardiography was performed using an M‐mode transducer (30 MHz phased‐array transducer, Vevo 1100, Canada) on day 7. At the papillary muscle level, the left ventricular end‐systolic diameter (LVESd) and LV end‐diastolic diameter (LVEDd) were measured by long‐axis views of M‐mode tracings. The LV ejection fraction (LVEF) and fractional shortening (FS) were calculated based on LVESd and LVEDd.

### 2.11 TUNEL staining

The heart tissues were harvested and embedded in optimal cutting temperature compound, and then, they were cut into approximately 5‐μm thick sections. The sections were firstly stained with cTNI and then were subjected to the TUNEL (Roche, Basel, Switzerland) staining to detect cell apoptosis. The section images were taken by a Nikon ECLIPSE Ti microscope (Olympus BX51, Japan).

### 2.12 Western blot analysis

Proteins were prepared from homogenized myocardial tissue and HCM cell samples. A BCA Protein Assay Kit (Beyotime, Songjiang, Shanghai, China) was used to determine the protein concentration. 20‐40 μg protein was separated by 8%‐12% polyacrylamide gel containing sodium dodecyl sulphate (SDS‐PAGE) and then was transferred onto the PVDF membrane (Thermo Fisher, Waltham, MA, USA). The membrane was blocked in 5% non‐fat milk at room temperature for 2 hours. Then, the membrane was incubated with primary antibodies as Table S2 at 4°C overnight. The membranes were incubated with a 1:2000 dilution of rabbit or mouse secondary antibodies (Cell Signaling Technology, Danvers, MA, USA) that were conjugated to the horseradish peroxidase at RT for 2 hours, and then were washed 10 minutes for 3 times. Finally, the membranes were detected with the enhanced chemiluminescence detection system (Millipore, MA).

### 2.13 mRFP‐GFP‐LC3 adenovirus transduction and confocal imaging

HCMs were transfected with Ad‐mRFP‐GFP‐LC3 (HanBio, China) at 50 MOI for 6 hours and then exposed to CVB3 at 10 MOI for 2 hours under FBS‐starvation condition to establish the cell VMC model. Then, cells were treated with or without exosomes (50 μg/mL) for next 24 hours. After that, the cells were washed, fixed with 4% paraformaldehyde and viewed under a laser scanning confocal microscope (Leica TCS SP5, Germany). The number of GFP and mRFP dots was determined by manual counting of fluorescent puncta. At least 40 cells were scored in each experiment. The number of dots per cell was obtained by dividing the total number of dots and the number of nuclei in each microscopic field.

### 2.14 Annexin V and PI assay for apoptosis ratio

HCMs were seeded into a 12‐well plate at a density of 1 × 10^5^ cells per well. Cells were incubated with exosomes (50 μg/mL) and supplemented CC (10 μmol/L) or RAPA (20 nmol/L). An Annexin V‐FITC/PI apoptosis detection kit (KeyGEN Biotech, Jiangning, Nanjing, China) was used to evaluate both infant and terminal cellular apoptosis under the manufacturer's instruction. All cells were collected by digestion with 0.25% trypsin, then washed twice with cold PBS and resuspended in annexin V‐binding buffer. Cells were further stained darkly with the mixture of 5 μL annexin V‐fluorescein isothiocyanate and 5 μL propidium iodide (PI) for 15 minutes at RT. The apoptotic cells including annexin V^+^/PI^‐^ were counted. DTX800 Multimode Detector (Beckman Coulter, CA) was used to determine fluorescence intensity and quantify the apoptosis ratio.

### 2.15 Statistical analysis

All data were expressed as mean ± SD. Comparisons of each group were evaluated using one‐way analysis of variance (ANOVA) followed by Dunnett's multiple comparison test. The differences in the pathological scores were performed by Mann‐Whitney U test. The differences between two groups were analysed using Student's unpaired t test. Statistical analyses were performed using SPSS 22.0 software. A value of *P* < 0.05 was considered statistically significant.

## 3 RESULTS

### 3.1 Identification of hucMSCs and exosomes derived from hucMSCs

**Figure 1:**
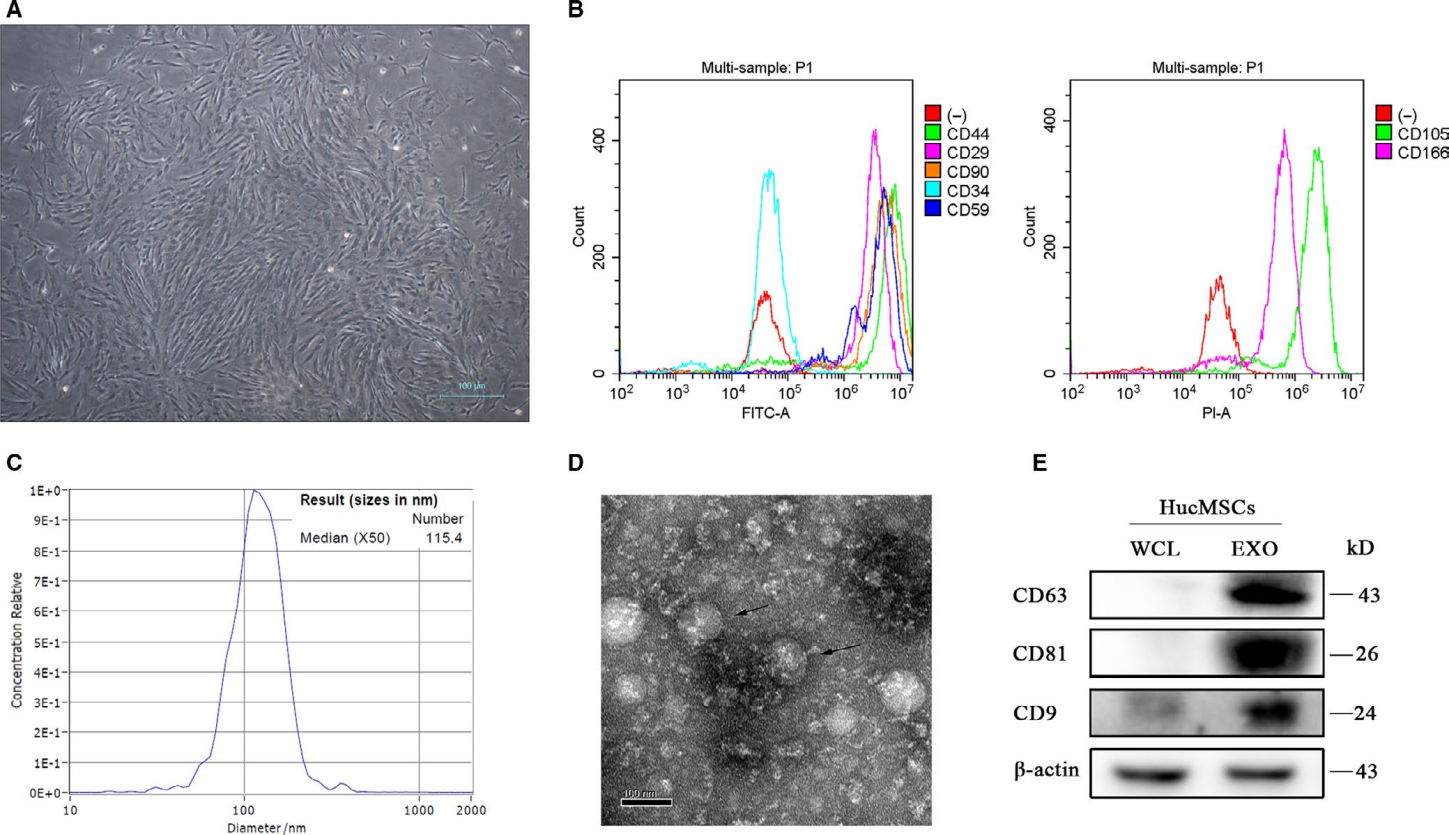
Identification of hucMSCs and hucMSC‐exosomes. A, The phase morphology of isolated hucMSCs. Scale bar, 100 μm. B, Flow cytometry analysed the expressions of MSC surface markers. C, The size distribution of hucMSC‐exosomes using nanoparticle tracking analysis (NTA). D, The size and shape of hucMSCs‐exosomes (black arrows) using transmission electron microscopy (TEM). Scale bar, 100 nm. E, Western blot analysis

hucMSCs isolated from human umbilical cords can display typical mesenchymal stem cell‐like spindle shape (Figure 1A). The results of flow cytometry showed that isolated huMSCs could positively express CD44, CD29, CD90, CD59, CD105 and CD166, but negatively express CD34 (Figure 1B), which were consistent with the previous report.^34^ The exosomes derived from huMSCs (hucMSC‐exosomes) were harvested through the ultracentrifugation. The result of nanoparticle tracking analysis (NTA) demonstrated that the size of hucMSC‐exosomes is ranged in a mono‐peak fashion and the average size is 115.4 nm (Figure 1C). In addition, the imaging of transmission electron microscopy (TEM) showed that hucMSC‐exosomes presented as cup base shape in diameter with about 100 nm (Figure 1D). Meanwhile, Western blot assay revealed that hucMSC‐exosomes could positively express the exosome special protein markers such as CD63, CD81 and CD9 (Figure 1E). These results indicated the isolated cells from human umbilical cord tissues were hucMSCs, and hucMSC‐exosomes were successfully isolated.

### 3.2 Uptake of hucMSC‐exosomes by heart tissue in vivo and HCMs in vitro

**Figure 2:**
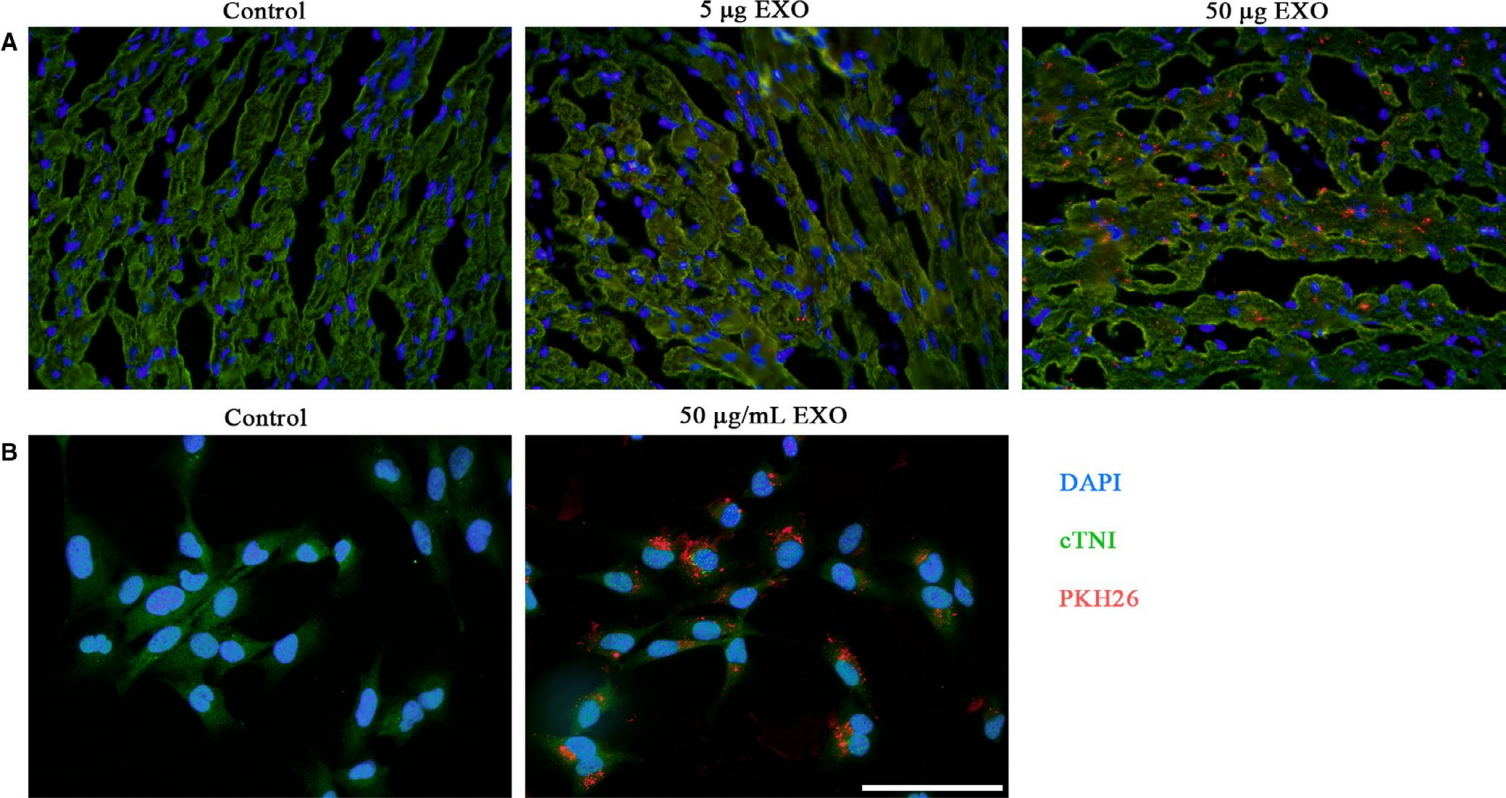
Uptake of hucMSC‐exosomes by heart tissues and HCMs. Fluorescence microscopy confirmed the location of hucMSC‐exosomes tagged with PKH26 (red fluorescence) in heart cardiomyocytes (A) and in cultured HCMs (B) stained with cTNI. Scale bar, 100 μm

In order to demonstrate hucMSC‐exosomes could be taken in by heart tissues in vivo and HCMs in vitro, hucMSC‐exosomes were firstly labelled with PKH26, a fluorescent cell linker compound, which shows strong red fluorescence under the fluorescence microscope. As shown in Figure 2A, hucMSC‐exosomes with red fluorescence could be successfully distributed into cardiomyocytes which were stained with cTNI, and the density of fluorescence became stronger with the increasing of hucMSC‐exosomes’ concentration. Moreover, hucMSC‐exosomes could also be endocytosed by HCMs, which were stained with cTNI (Figure 2B). In contrast, the control group in vivo or in vitro, there was no any intracellular red fluorescence. These results suggested that hucMSC‐exosomes could be taken in by heart tissue and HCMs.

### 3.3 Effects of hucMSC‐exosomes on the heart function of CVB3‐induced myocarditis

**Figure 3:**
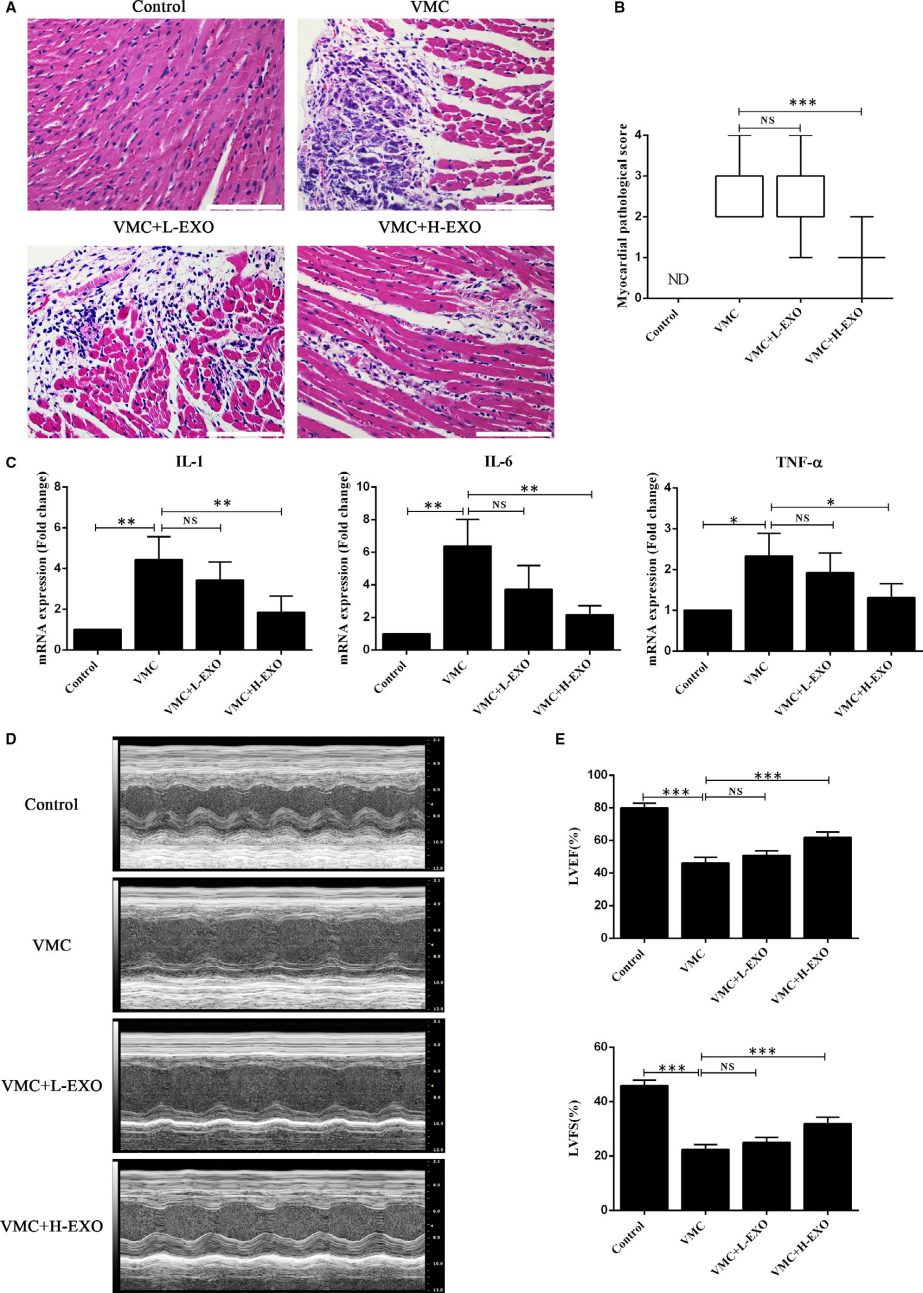
Effects of hucMSC‐exosomes on the heart function of CVB3‐induced myocarditis. A, H&E staining in the heart tissues. Scale bar, 100 μm. B, The histopathological scores of each group (n = 8). C, The expressions of IL‐1, IL‐6 and TNF‐α in the heart tissues (n = 6) using qPCR assay. D, The typical M‐mode echocardiograms (n = 8). E, The quantitative results of LVEF and LVFS in these four groups. Data were expressed as mean ± SD. NS means no significance differences. *P < 0.05, **P < 0.01, ***P < 0.001 indicate significant differences when compared with control (0.1 mL NS without CVB3) or hucMSC‐exosome treatment (0 μg), respectively

To explore the function of hucMSC‐exosomes on coxsackievirus B3 (CVB3)‐induced myocarditis (VMC), the low concentration hucMSC‐exosomes (5 μg, L‐EXO) and high concentration (50 μg, H‐EXO) were injected into the VMC mice. The result of H&E staining showed that hucMSC‐exosomes (50 μg, H‐EXO) could effectively rescue the cellular infiltration and necrosis of VMC mice (Figure 3A) as well as significantly improve the myocardial pathological score (Figure 3B). However, hucMSC‐exosomes (5 μg, L‐EXO) had no remarkable performance. In addition, the data of qPCR showed that hucMSC‐exosomes in H‐EXO but not L‐EXO could distinctly decrease the expressions of proinflammatory cytokines such as IL‐1, IL‐6 and TNF‐α in heart tissues of VMC mice (Figure 3C). Furthermore, the M‐mode image showed hucMSC‐exosomes in H‐EXO but not L‐EXO could also improve the wall motion of VMC mice (Figure 3D). The quantitative result illuminated that hucMSC‐exosome administration in H‐EXO could significantly increase the values of LVEF and LVFS in VMC mice (Figure 3E). These results demonstrated that hucMSC‐exosomes (50 μg, H‐EXO) could effectively rescue the heart function of CVB3‐induced myocarditis mice.

### 3.4 HucMSC‐exosomes attenuated cell apoptosis in CVB3‐induced myocarditis mice

**Figure 4:**
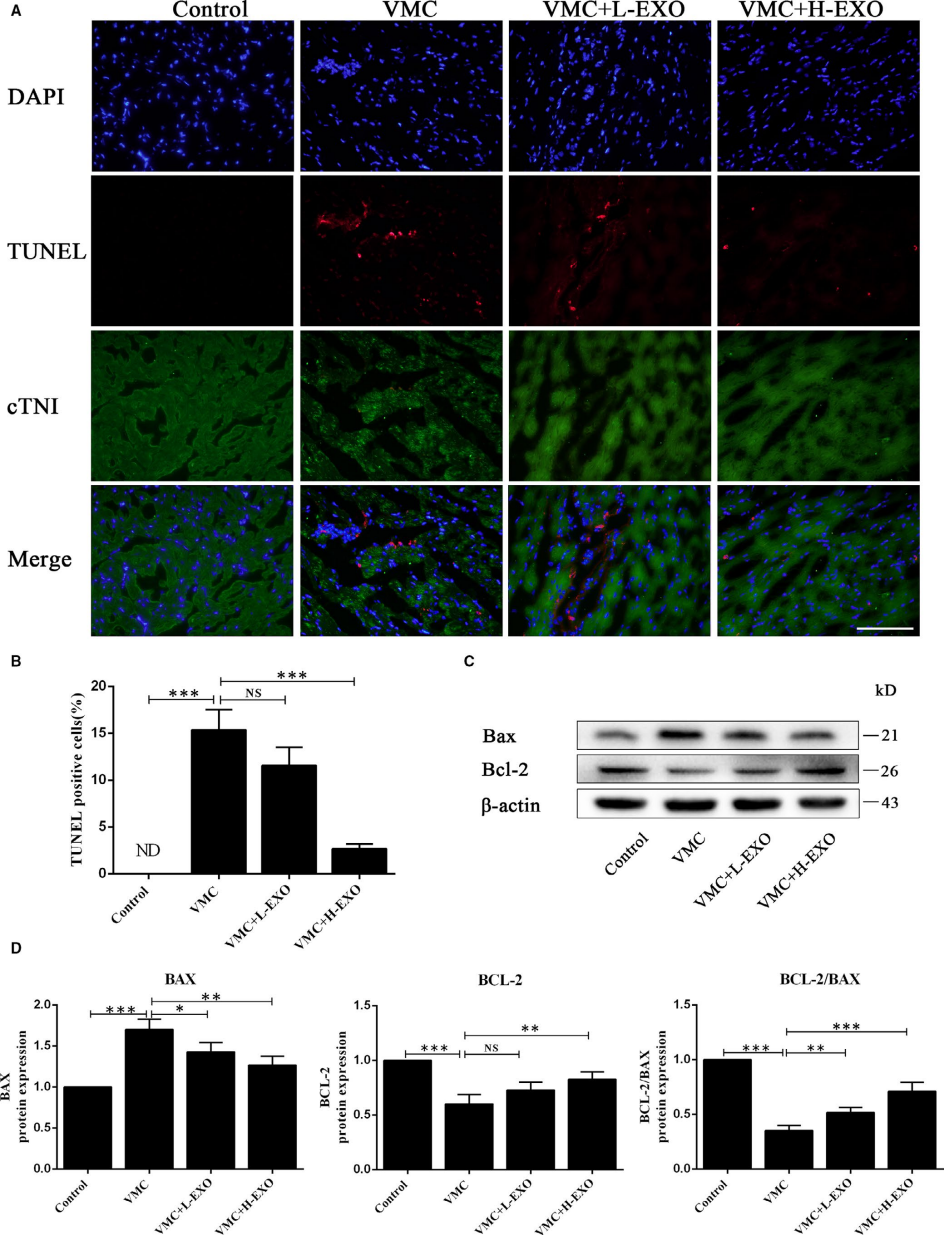
hucMSC‐exosomes attenuated the apoptosis in VMC in vivo*.* A, TUNEL staining in the heart tissues stained with cTNI. Scale bar, 100 μm. B, The percentages of TUNEL‐positive cells. C, Western blot analysis. D, The quantitative results of Western blot. Data were expressed as mean ± SD. n = 6. NS means no significance differences. *P < 0.05, **P < 0.01, ***P < 0.001 indicate significant differences when compared with control (0.1 mL NS without CVB3) or hucMSC‐exosome treatment (0 μg), respectively

Apoptosis is an important index of myocarditis.^35^, ^36^ In the heart tissues of CVB3‐induced myocarditis mice, there were a lot of apoptosis cells comparing with the normal control group through TUNEL staining. However, hucMSC‐exosome (50 μg, H‐EXO) administration could effectively attenuate cell apoptosis of CVB3‐induced myocarditis mice (Figure 4A), and the quantitative result of TUNEL staining also showed that hucMSC‐exosome in H‐EXO but not in L‐EXO could significantly reduce the percentage of TUNEL‐positive cells in CVB3‐induced myocarditis mice (Figure 4B), although hucMSC‐exosome (5 μg, L‐EXO) had slight effect. Moreover, the results of Western blot showed that hucMSC‐exosomes in H‐EXO and L‐EXO could distinctly inhibit the expression of apoptosis protein BAX in CVB3‐induced myocarditis mice, but just hucMSC‐exosome (50 μg, H‐EXO) could enhance the anti‐apoptosis protein Bcl‐2 in CVB3‐induced myocarditis mice (Figure 4C,D). These data suggested that hucMSC‐exosome (50 μg, H‐EXO) could inhibit cell apoptosis in CVB3‐induced myocarditis mice.

### 3.5 HucMSC‐exosomes activated AMPK/mTOR‐mediated autophagy flux pathway in CVB3‐induced myocarditis in vivo and in vitro

**Figure 5:**
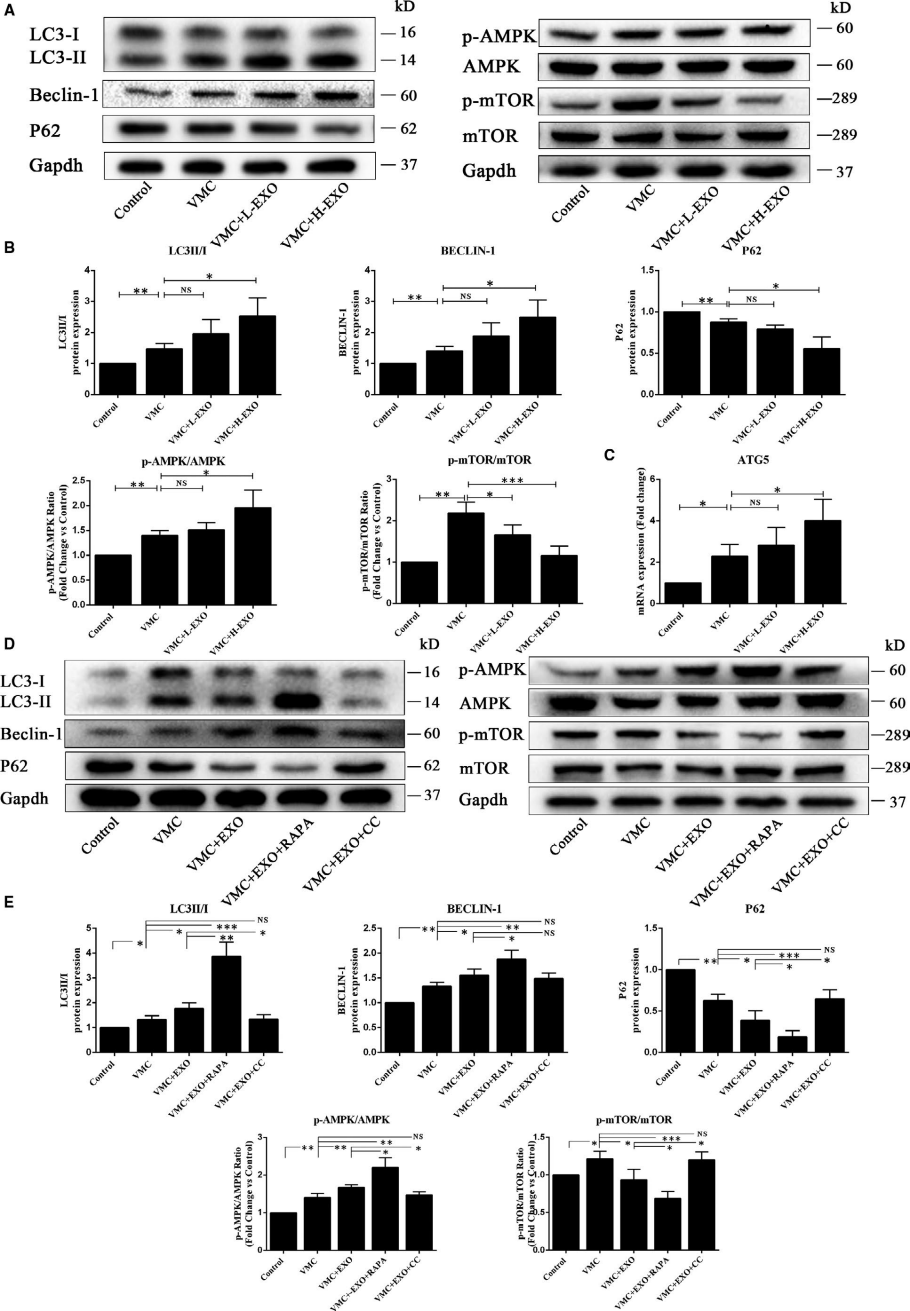
hucMSC‐exosomes activated autophagy flux in vivo and in vitro. A, Western blot analysis of protein expressions in heart tissues in vivo. B, The quantitative results of Western blot. C, The expression of ATG5 gene in the heart tissues using qPCR assay. D, Western blot analysis of protein expressions in HCMs in vitro. E, The quantitative results of Western blot. Data were expressed as mean ± SD. n = 6. NS means no significance differences. *P < 0.05, **P < 0.01, ***P < 0.001 indicate significant differences when compared with control (0.1 mL NS without CVB3 or 0 MOI CVB3) or hucMSC‐exosome treatment (0 or 50 μg/mL), respectively

To explore the potential molecular mechanisms involved in the effects of hucMSC‐exosomes on CVB3‐induced myocarditis in vivo and in vitro, the relevant protein expressions of AMPK/mTOR‐mediated autophagy flux pathway were investigated by Western blot (Figure 5A,D). The quantitative results in vivo showed that hucMSCs‐exosomes in H‐EXO (50 μg) could significantly increase the levels of pAMPK, autophagy proteins LC3‐II/I and Beclin‐1 but decrease the level of pmTOR and promote the degradation of autophagy flux marker P62, without affecting AMPK and mTOR levels in the heart tissues of CVB3‐induced myocarditis mice (Figure 5B). In addition, the quantitative results in vitro also showed that hucMSC‐exosomes (50 μg/mL) could up‐regulate the expressions of pAMPK, LC3II/I and Beclin‐1 but down‐regulate the level of pmTOR and promote the degradation of P62 in the HCMs with CVB3 treatment. Moreover, the autophagy agonist rapamycin (RAPA) further enhanced the levels of pAMPK, LC3II/I and BECLIN‐1 but reduced the levels of pmTOR and P62 when compared with VMC + EXO group. However, AMPK inhibitor compound c (CC) effectively reversed this trend (Figure 5E). These results suggested that hucMSC‐exosomes effectively activated the AMPK/mTOR‐mediated autophagy flux pathway in CVB3‐induced myocarditis.

**Figure 6:**
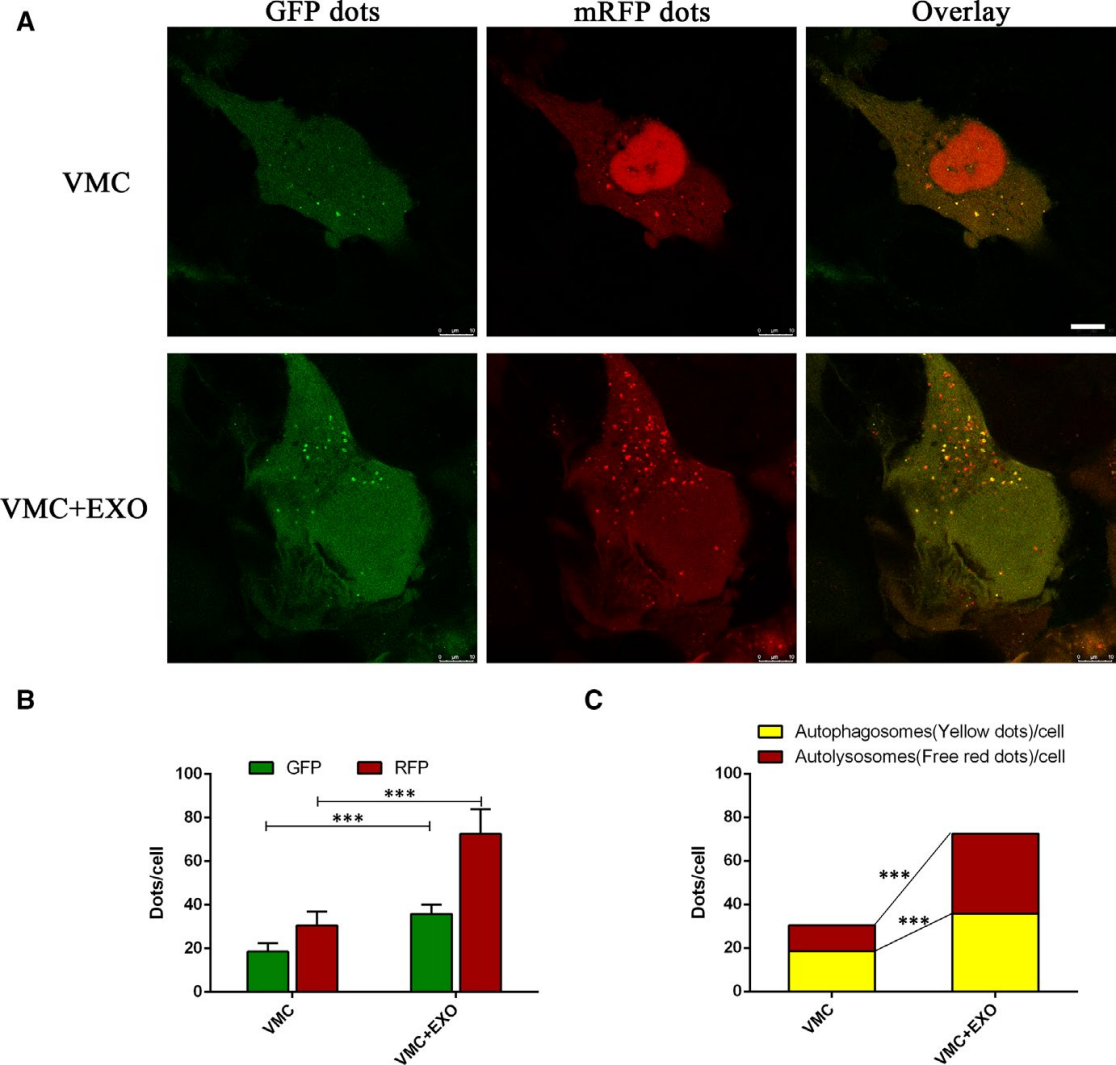
hucMSC‐exosomes enhanced autophagy flux in CVB3‐induced myocarditis in vitro. A, The confocal imaging of HCMs with CVB3 treatment after Ad‐mRFP‐GFP‐LC3 transfection. Scale bar, 10 μm. B, The quantitative analysis of GFP dots and mRFP dots. Green dots represent autophagosomes, and red dots represent autophagosomes and autolysosomes. C, The quantitative analysis of merged images with both yellow dots (autophagosomes) and red dots (autolysosomes). Data were expressed as mean ± SD. n = 6. ***P < 0.001 indicates significant differences when compared with control (VMC)

In order to accurately reflect the process of autophagy, Ad‐mRFP‐GFP‐LC3 transfection method was used to detect the effects of hucMSC‐exosomes on autophagy flux in HCMs with CVB3 treatment (Figure 6A). GFP dots (green) represent autophagosomes, and mRFP dots (red) represent autophagosomes and autolysosomes. The quantitative results showed that the treatment of huMSC‐exosomes could significantly increase the numbers of GFP and mRFP dots compared with VMC group (Figure 6B). The mean number of autophagosomes was based on dots with both red and green displaying yellow in merged images, and the mean number of autolysosomes was assessed dots with only red but not green in merged images. The quantitative results of merged images showed that hucMSC‐exosomes also significantly increased both yellow dots (autophagosomes) and red dots (autolysosomes) (Figure 6C). Meanwhile, the qPCR results also showed that huMSC‐exosomes (50 μg, H‐EXO) significantly up‐regulated the level of ATG5 gene in VMC in vivo, which plays an important role in the extension of autophagosome membrane and autophagy formation (Figure 5C). These results suggested that hucMSC‐exosomes could enhance autophagy and autophagic flux in CVB3‐induced myocarditis.

### 3.6 HucMSC‐exosomes attenuated cell apoptosis in CVB3‐induced HCMs

**Figure 7:**
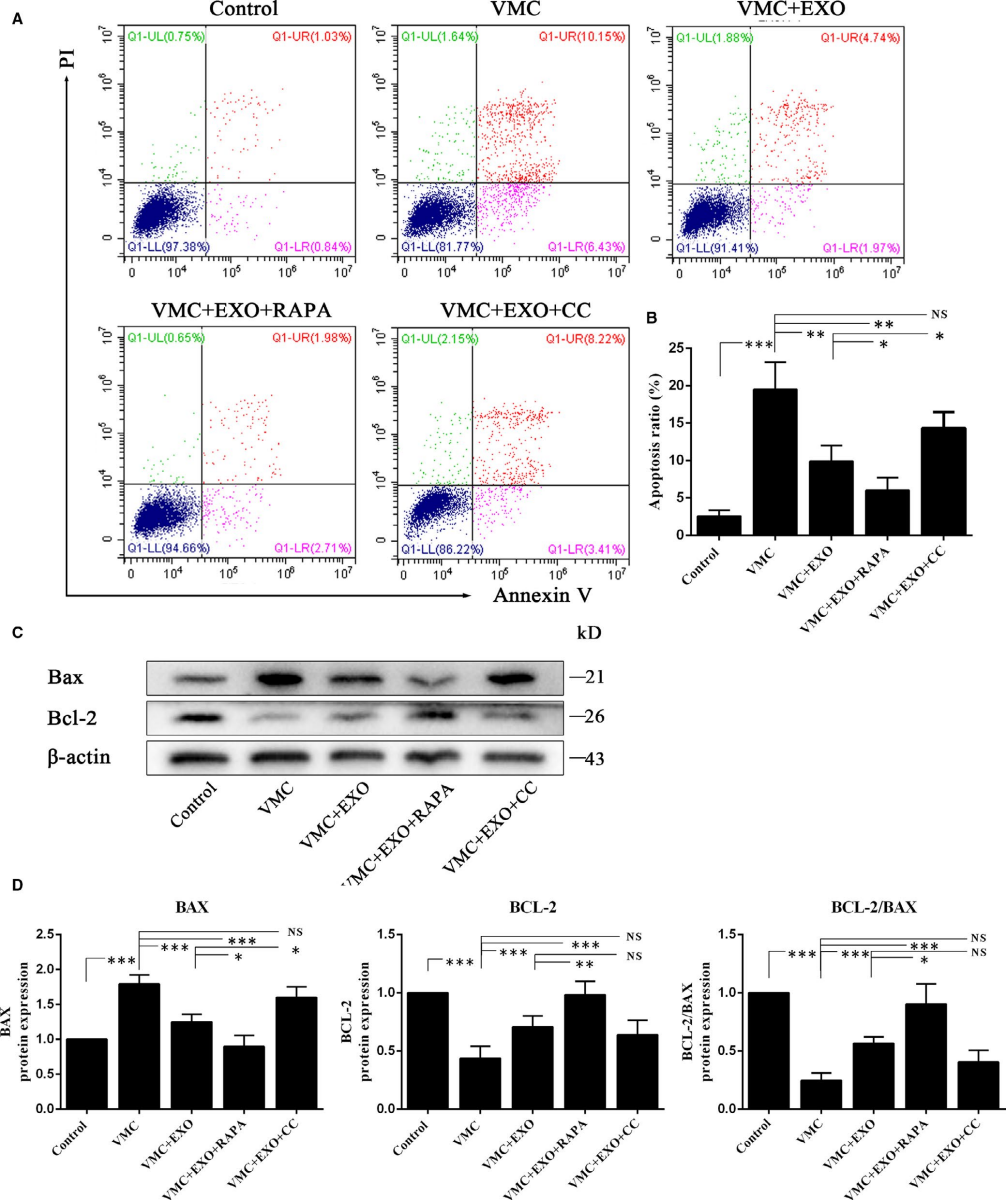
hucMSC‐exosomes attenuated cells apoptosis in CVB3‐induced myocarditis in vitro. A, Detection the cell apoptosis using annexin V and PI assay. B, The quantitation of annexin V and PI assay. C, Western blot analysis. D, The quantitation of Western blot. Data were expressed as mean ± SD. n = 6. NS means no significance differences. *P < 0.05, **P < 0.01, ***P < 0.001 indicate significant differences when compared with control (0 MOI CVB3) or hucMSC‐exosome treatment (0 or 50 μg/mL), respectively

To further confirm that hucMSC‐exosomes might prevent CVB3‐induced cell apoptosis, we detected the cell apoptosis of HCM with different treatments through flow cytometry (Figure 7A) and Western blot (Figure 7C). The percentages of apoptotic cells were calculated from the late apoptosis (Q1‐UR) and early apoptosis (Q1‐LR). The data of annexin V and PI assay showed that the apoptosis ratio in the control group was just 1.87%, but in CVB3‐induced HCMs (VMC) was rushed to 16.58%. hucMSC‐exosomes (50 μg/mL) could partially inhibit CVB3‐induced HCM apoptosis with a rate of 6.71%, and RAPA further reduced cell apoptosis with a rate of 4.69% but CC significantly enhanced cell apoptosis with a rate of 11.65% (Figure 7B). Moreover, the results of Western blot showed that the level of apoptosis protein BAX was remarkably up‐regulated but the expression of anti‐apoptosis protein BCL‐2 was distinctly down‐regulated in VMC group. hucMSC‐exosomes (50 μg/mL) could prevent CVB3‐induced HCM apoptosis through decreasing BAX expression and increasing BCL‐2 expression, which leaded to the higher ratio of BCL‐2/BAX. Meanwhile, RAPA could further enhance this trend to inhibit cell apoptosis but CC still reversed this trend (Figure 7D). These data suggest that hucMSC‐exosomes could inhibit cell apoptosis through activating AMPK/mTOR‐mediated autophagy flux pathway.

## 4 DISCUSSION

In the present study, we successfully isolated human umbilical cord mesenchymal stem cells (hucMSCs) and identified exosomes derived from hucMSCs (hucMSC‐exosomes). Our results showed that the administration of hucMSC‐exosomes following CVB3‐induced myocarditis significantly decreased myocardial pathological injury and proinflammatory cytokines as well as increased LVEF and LVFS. Moreover, hucMSC‐exosomes could significantly inhibit cell apoptosis through the activation of AMPK/mTOR‐mediated autophagy flux pathway. In a word, hucMSC‐exosome treatment has a protective effect against CVB3‐induced myocarditis (VMC).

VMC is an inflammatory disease in the myocardium with inflammatory infiltration, myocyte degeneration and necrosis of non‐ischaemic origin, leading to cardiac dysfunction.^1^ Inhibition of the inflammatory response and cell apoptosis after CVB3‐induced myocarditis could rescue cardiac dysfunction.^37^ Many studies had already demonstrated that exosomes could markedly influence the anti‐inflammatory response.^38^, ^39^, ^40^ Cosenza et al reported that exosomes derived from MSCs showed a dose‐dependent anti‐inflammatory effect in delayed‐type hypersensitivity, and decreased clinical signs of inflammation in collagen‐induced arthritis.^38^ Zhao et al showed that exosomes derived from adipose‐derived stem cells (ADSCs) drove alternatively activated M2 macrophage polarization and inflammation reduction.^39^ HucMSC‐exosomes could mediate miR‐181c attenuating burn‐induced inflammation.^40^ Our present study also demonstrated that hucMSC‐exosome administration not only reduced the inflammatory infiltration and the expressions of inflammatory cytokines (IL‐1, IL‐6 and TNF‐α) but also improved the cardiac function in VMC mice. CVB3‐induced myocarditis mice showed serious inflammation, increased proinflammatory cytokines and had cardiac dysfunction with lower ejection fraction and fractional shortening comparing with the normal control mice. These abnormalities could be effectively ameliorated by hucMSC‐exosome treatment.

In addition to anti‐inflammatory effects, exosomes also have anti‐apoptotic effects. Liao et al found that MSC‐exosomes can attenuate endoplasmic reticulum stress‐induced apoptosis by activating AKT and ERK signalling pathway in human nucleus pulposus cells. Exosomes also modulated apoptosis and retarded intervertebral disc degeneration progression in a rat model.^41^ Moreover, M2 microglia‐derived exosomes could attenuate neural deficits and neuronal apoptosis in mice after stroke through containing miR‐124, which targeted the ubiquitin‐specific protease 14.^42^ Pan et al showed that delayed remote ischaemic preconditioning conferred renoprotection against septic acute kidney injury via containing miR‐21, which mediated downstream PDCD4/NF‐κB and PTEN/AKT pathways to exert anti‐inflammatory and anti‐apoptotic effects.^43^ In this study, we also found that hucMSC‐exosomes could reduce apoptosis in CVB3‐induced myocarditis in vivo and in vitro. Furthermore, hucMSC‐exosomes significantly decreased the expressions of apoptotic protein Bax and increased the levels of anti‐apoptotic protein Bcl‐2 in VMC mice and HCMs subjected to CVB3.

The functional relationship between apoptosis and autophagy is complex. Under some conditions, autophagy could exhibit a stress adaptation that prevents cell death and suppresses apoptosis.^44^ Autophagy, cellular self‐digestion, is a lysosomal degradation pathway involved in protein and organelle degradation, with a number of connections to human disease and physiology.^45^, ^46^ Autophagy principally plays an adaptive role to protect organisms against diverse pathologies including infection, cancer, neurodegeneration, ageing and heart diseases.^45^, ^47^ Microtubule‐associated protein light chain 3 (LC3) existing in autophagosome is essential for autophagosome formation and servers as a marker for autophagosome.^48^, ^49^ Beclin‐1 is also important in the initiation of autophagy, mediating the localization of other autophagy proteins.^50^ Therefore, the increased levels of LC3 and Beclin‐1 indicate the autophagy activation. ATG5 gene plays an important role in the extension of autophagosome membrane and autophagy formation.^49^ In our study, hucMSC‐exosome treatment significantly enhanced the level of ATG5 gene in VMC in vivo. P62, an adaptor protein with multiple protein‐protein interaction domains, is selectively incorporated into autophagosomes through binding to LC3 and ubiquitinated cargo, and is efficiently degraded by autophagy.^51^, ^52^ Thus, the level of P62 is inversely correlated with autophagic activity.^49^ Autophagic flux is a dynamic and multistep process that involves the formation of autophagosomes, the fusion of autophagosomes with lysosomes and its subsequent breakdown and release of the resulting macromolecules back into the cytosol.^53^ In this study, CVB3 induced autophagy as the same as our previous study.^37^ HucMSC‐exosome administration further significantly increased the expressions of autophagy biomarkers LC3II/I and Beclin‐1, and promoted the degradation of P62 in VMC in vivo and vitro. In addition, combining with the results of Ad‐mRFP‐GFP‐LC3 transfection, these results indicated that hucMSC‐exosomes could activate autophagy flux through activating the degradation of P62, augmenting the formation of LC3 and Beclin‐1, and up‐regulating ATG5 gene expression.

**Figure 8:**
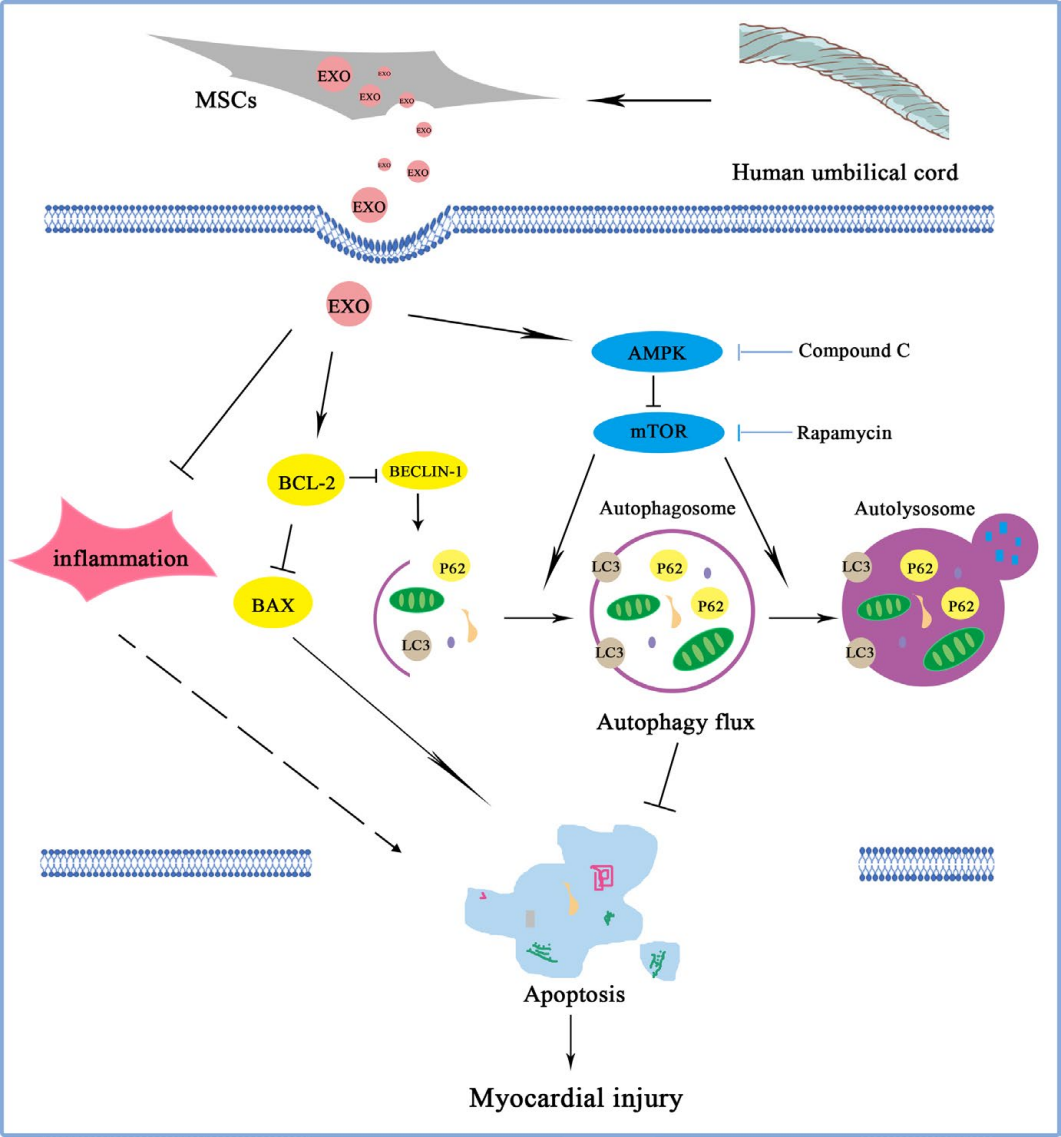
Diagram of the potential mechanisms involved in the effects of hucMSC‐exosomes on CVB3‐induced myocarditis

Many signalling pathways can participate in the autophagy regulation. AMPK is a sensor of the overall cellular energy charge that regulates cellular metabolism to maintain energy homeostasis and generally induces autophagy.^53^, ^54^ mTOR, a downstream agent of AMPK, is also involved in the regulation of autophagy.^55^ Inhibition of mTOR can promote autophagy, and the activation of AMPK is antagonistic towards mTOR function.^56^ In diabetic OVE26 mice, AMPK activation by metformin could prevent cardiomyopathy by up‐regulating autophagy activity.^57^ Ablation of mTOR increased autophagy signalling and reduced the degradation and apoptosis of articular cartilage.^58^ In this study, we also found that hucMSC‐exosomes could activate the AMPK/mTOR pathway through increasing pAMPK/AMPK ratio and decreasing pmTOR/mTOR ratio. Spermidine‐enhanced autophagic flux improved cardiac dysfunction by activation of AMPK/mTOR signalling pathway in myocardial infarction.^59^ Exosomes secreted from adipose‐derived stem cells could attenuate diabetic nephropathy through promoting autophagy flux and inhibiting apoptosis in podocyte.^60^ Our results showed that AMPK inhibitor CC distinctly reduced pAMPK/AMPK ratio and enhanced pmTOR/mTOR level comparing with VMC + EXO group. Moreover, AMPK inhibitor CC could also up‐regulate the levels of P62 and Bax, and increase the apoptosis ratio as well as down‐regulate LC3II/I ratio comparing with VMC + EXO group. However, autophagy agonist RAPA augmented the activation of hucMSC‐exosomes on the AMPK/mTOR pathway through increasing the levels of LC3II/I, Bcl‐2/Bax, Beclin‐1 and Bcl‐2, promoting the degradation of P62, decreasing the expressions of Bax and reducing the apoptosis ratio. These data demonstrated that hucMSC‐exosomes might inhibit cell apoptosis through activating AMPK/mTOR‐mediated autophagy flux pathway. The detailed diagram of mechanism involved in the effects of hucMSC‐exosomes on CVB3‐induced myocarditis is shown in Figure 8.

As is well known, the exosomes contain proteins such as cytoskeletal proteins, signal transduction‐related proteins, metabolic enzymes, antigen binding presents related proteins and nucleic acids like mRNA, miRNA, lncRNA, mtDNA and circRNA.^61^, ^62^ Some proteins or nucleic acids have homologous functions in different species. The proteins or RNAs carried by hucMSC‐exosomes may be associated with the promotion of cell proliferation and suppression cell apoptosis. It has been reported that cardiomyocytes and endothelial cells could take in exosomes to protect the cells from injury.^63^, ^64^ Jin et al reported that exosomes derived from adipose‐derived stem cells ameliorated diabetic nephropathy symptom through enhancing the expression of miR‐486, which led to the inhibition of Smad1/mTOR signalling pathway in podocyte.^60^ Exosome‐transmitted LINC00461 could promote multiple myeloma cell proliferation and suppress apoptosis by modulating microRNA/BCL‐2 expression.^65^ Exosomes with abundant miR‐100‐5p derived from infrapatellar fat pad MSCs could protect articular cartilage and ameliorate gait abnormalities by inhibition of mTOR expression in osteoarthritis.^66^ As we all know, exosomes contain many RNAs and proteins. In the present study, we did not clearly illuminate the specific mediators in exosomes that is the limitation of this study, so we should further explore in the future.

In summary, this study demonstrated that hucMSC‐exosomes could exert the partially protective effects on CVB3‐induced myocarditis by activating AMPK/mTOR‐mediated autophagy flux pathway to attenuate cells apoptosis.

## CONFLICT OF INTEREST

The authors declare that they have no conflict of interest.

## AUTHOR CONTRIBUTIONS

X.Guo, JL and MC conceived and designed research; X.Gu, YL, KC, XW, ZW, HL, YL and XR performed experiments; X.Gu, YL, X.Guo, JL and MC analysed data and interpreted results of experiments; X.Gu drafted manuscript; X.Guo, JL and MC edited and revised manuscript; X.Guo and X.Gu approved final version of manuscript.

## Supporting information

Table S1

Table S2

Table S3

## Data Availability

Data are available on request from the authors.
