# Supplementary material for: Exosomes derived from umbilical cord mesenchymal stem cells alleviate viral myocarditis through activating AMPK/mTOR‐mediated autophagy flux pathway
Source: J Cell Mol Med. 2020 May 18;24(13):7515–30. doi: 10.1111/jcmm.15378 (PMC7339183; doi:10.1111/jcmm.15378)
Supplement: Supplementary file 1 — Table S1 [file JCMM-24-7515-s001.doc]

**Supplementary Table 1. Flow cytometry antibodies**

| **Antibody** | **Species** | **Company (catalogue)** | **Dilution** |  |
| --- | --- | --- | --- | --- |
| CD44-FITC | Mouse | eBioscience (11-0441-82) | 1:100 | |
| CD29-FITC | Mouse | eBioscience (11-0291-82) | 1:20 | |
| CD90-FITC | Mouse | eBioscience (11-0909-42) | 1:20 | |
| CD34-FITC | Mouse | eBioscience (11-0341-82) | 1:50 | |
| CD59-FITC | Mouse | eBioscience (11-0596-42) | 1:20 | |
| CD105-PE | Mouse | eBioscience (12-1057-42) | 1:20 | |
| CD166-PE | Mouse | eBioscience (12-1661-82) | 1:20 | |
| IgG1 isotype control-FITC | Mouse | ThermoFisher (GM4992) | 1:20 | |
| IgG1 isotype control-PE | Mouse | ThermoFisher (GM4993) | 1:20 | |
