# Supplementary material for: Exosomes derived from umbilical cord mesenchymal stem cells alleviate viral myocarditis through activating AMPK/mTOR‐mediated autophagy flux pathway
Source: J Cell Mol Med. 2020 May 18;24(13):7515–30. doi: 10.1111/jcmm.15378 (PMC7339183; doi:10.1111/jcmm.15378)
Supplement: Supplementary file 2 — Table S2 [file JCMM-24-7515-s002.doc]

**Supplementary Table 2. Antibodies**

| **Antibody** | **Species** | **Company (catalogue)** | **Dilution** | |
| --- | --- | --- | --- | --- |
|  |  |  | **WB** | **IHC/IF** |
| CD81 | Mouse | [Santa Cruz Biotechnology](http://www.baidu.com/link?url=JyKcE01MHDo2C82_vA7PLGsOo6FHhidzFLsl0vS1U__) (sc-7637) | 1:200 | 1:50 |
| CD63 | Mouse | [Santa Cruz Biotechnology](http://www.baidu.com/link?url=JyKcE01MHDo2C82_vA7PLGsOo6FHhidzFLsl0vS1U__) (sc-5275) | 1:200 | 1:50 |
| CD9 | Mouse | [Santa Cruz Biotechnology](http://www.baidu.com/link?url=JyKcE01MHDo2C82_vA7PLGsOo6FHhidzFLsl0vS1U__) (sc-13118) | 1:200 | 1:50 |
| GAPDH | Rabbit | Cell Signaling Technology (#5174) | 1:1000 | ND |
| βACTIN | Rabbit | Cell Signaling Technology (#4970) | 1:1000 | ND |
| AMPK | Rabbit | Cell Signaling Technology (#5831) | 1:1000 | ND |
| pAMPK | Rabbit | Cell Signaling Technology (#50081) | 1:1000 | ND |
| mTOR | Rabbit | Cell Signaling Technology (#2983) | 1:1000 | ND |
| pmTOR | Rabbit | Cell Signaling Technology (#5536) | 1:1000 | ND |
| BECLIN-1 | Rabbit | Abcam (ab207612) | 1:1000 | ND |
| LC3 | Rabbit | Cell Signaling Technology (#4108) | 1:1000 | 1:50 |
| P62 | Mouse | Abcam (ab56416) | 1:1000 | 1:100 |
| BAX | Rabbit | Abcam (ab32503) | 1:1000 | ND |
| BCL-2 | Rabbit | Cell Signaling Technology (#3498) | 1:1000 | ND |
| cTNI | Rabbit | Abcam (ab47003) | 1:1000 | 1:100 |

ND = Not detected; WB = Western blot; IHC: Immunohistochemistry; IF: Immunofluorescence.
