# Supplementary material for: Exosomes derived from umbilical cord mesenchymal stem cells alleviate viral myocarditis through activating AMPK/mTOR‐mediated autophagy flux pathway
Source: J Cell Mol Med. 2020 May 18;24(13):7515–30. doi: 10.1111/jcmm.15378 (PMC7339183; doi:10.1111/jcmm.15378)
Supplement: Supplementary file 3 — Table S3 [file JCMM-24-7515-s003.doc]

**Supplementary T**able 3. Primer information

| **Primer**  **Symbol** | **Gene name** | **Primer direction** | **Sequences (5’to 3’)** | **PCR**  **(bp)** | **Accession** |
| --- | --- | --- | --- | --- | --- |
| *IL-1* | Interleukin 1 | Forward | TTCATCTCGGAGCCTGTAGTG | 158 | NM_05334 |
|  |  | Reverse | TGTCTTTCCCGTGGACCTT |  |  |
| *IL-6* | Interleukin 6 | Forward | CACCAGCATCAGTCCCAAG | 99 | NC_000071.6 |
|  |  | Reverse | GGAGCCCACCAAGAACG |  |  |
| *TNF-α* | Tumour necrosis factor alpha | Forward | GCTCCTCCACTTGGTGGTTTGT | 186 | NC_000083.6 |
|  |  | Reverse | ACTCCAGGCGGTGCCTATGTC |  |  |
| *ATG5* | Autophagy related 5 | Forward | ATCCAAGGATGCGGTTGAGG | 117 | NM_001314013 |
|  |  | Reverse | ATCCAGAGCTGCTTGTGGTC |  |  |
| *GAPDH* | Glyceraldehyde-3-phosphate dehydrogenase | Forward | GGTTGTCTCCTGCGACTTCA | 111 | NC_000072.6 |
|  |  | Reverse | TGGTCCAGGGTTTCTTACTCC |  |  |
